# The Use of Peptides in Veterinary Serodiagnosis of Infectious Diseases: A Review

**DOI:** 10.3390/vetsci9100561

**Published:** 2022-10-12

**Authors:** Saúl Aguilar-Montes de Oca, Roberto Montes-de-Oca-Jiménez, Juan Carlos Vázquez-Chagoyán, Alberto Barbabosa-Pliego, Pilar Eliana Rivadeneira-Barreiro, Pablo C. Zambrano-Rodríguez

**Affiliations:** 1 Centro de Investigación y Estudios Avanzados en Salud Animal, Facultad de Medicina Veterinaria y Zootecnia, Universidad Autónoma del Estado de México, Carretera Toluca-Atlacomulco, Km 15.5, Toluca 50200, CP, Mexico; 2 Departamento de Veterinaria, Facultad de Ciencias Veterinarias, Universidad Técnica de Manabí, Portoviejo 130105, Ecuador

**Keywords:** synthetic peptides, pELISA, veterinary diagnosis, disease, epitopes

## Abstract

**Simple Summary:**

The diagnosis of several animal diseases is critical when animal health is threatened and can cause a serious financial loss, although different diagnostic tools implemented are not always affordable or easy to perform. Serological diagnosis is successful in this aspect, however, there are not always suitable antigens, or these show low levels of sensitivity and specificity. Some studies with peptides applied to serological diagnoses that have been implemented in recent years with their respective observations are explored. The use of small sequences that can be synthesized and customized is attractive and functional in some diseases from domestic animals and wildlife. This review highlights the possibilities and limitations of peptides and will help researchers and people interested in implementing a diagnostic system to decide between them for several animal diseases.

**Abstract:**

Peptides constitute an alternative and interesting option to develop treatments, vaccines, and diagnostic tools as they demonstrate their scope in several health aspects; as proof of this, commercial peptides for humans and animals are available on the market and used daily. This review aimed to know the role of peptides in the field of veterinary diagnosis, and include peptide-based enzyme-linked immunosorbent assay (pELISA), lateral flow devices, and peptide latex agglutination tests that have been developed to detect several pathogens including viruses and bacteria of health and production relevance in domestic animals. Studies in cattle, small ruminants, dogs, cats, poultry, horses, and even aquatic organisms were reviewed. Different studies showed good levels of sensitivity and specificity against their target, moreover, comparisons with commercial kits and official tests were performed which allowed appraising their performance. Chemical synthesis, recombinant DNA technology, and enzymatic synthesis were reviewed as well as their advantages and drawbacks. In addition, we discussed the intrinsic limitations such as the small size or affinity to polystyrene membrane and mention several strategies to overcome these problems. The use of peptides will increase in the coming years and their utility for diagnostic purposes in animals must be evaluated.

## 1. Introduction

The attentiveness to the potential of peptides has boosted their use for several applications: vaccine development [1,2], anti-cancer treatment [3], regulation of the immune system [4], diagnosis [5], and different treatments [6]; nowadays, several of them are marketed for human use, however, peptide-based products for Veterinary Medicine are also available.

The history of marketed peptides dates back to the 1970s and 1980s and new peptides are reviewed by federal agencies such as the Food and Drug Administration (FDA) and the European Medicines Agency (EMA); for example, from 2016 to 2020 a total of 17 peptide-based drugs for human use were approved by the FDA. It is important to note that most of these have been produced by chemical synthesis which is the preferred process, followed by recombinant technology; both technologies are discussed later in this review [7,8,9]. 

Comparably, several peptide-based drugs for Veterinary use are listed in the Green Book published by the FDA, some of them share a usage akin to those in humans, while others have particular applications in animals, especially hormonal peptides [10,11] (Table 1).

Peptide-based drug research is an emerging field with investigations published every year and the potential to find useful biomolecules of animals origin (or other sources) is almost boundless considering the enormous diversity of animal species that can be studied; animal sources of food such as eggs or milk, different tissues, or even animal venoms are promising sources of peptides [12,13,14]. Additionally, peptide studies with positive results have been performed in animal models to ensure their safety [15,16]; this allows us to speculate that an analog therapy with similar outcomes could be possible in domestic animals. 

In Veterinary Medicine, peptides have been investigated and used majorly in the field of animal nutrition, therapeutics, reproduction, vaccines, and diagnostic tools [17]. One of its most widely known uses is to treat infectious diseases caused by pathogens. Bacitracin, polymyxins B and E (colicin), fosfomycin, vancomycin, streptogramins (virginiamycin), and others are sundry examples of antimicrobial peptides (AMP) used in animals; most of them are obtained from different microorganisms, but also can be semisynthetic [11,18]. In the same way, when the antimicrobial resistance (AMR) phenomenon forced the ban of antibiotics as growth promoters in animal feed, the necessity to find a substitute allowed AMP to be tested as alternative feed additives [19,20,21].

## 2. Peptides in Diagnosis

A current review showed that the use of peptides in humans is focused mainly on three areas: drug development, diagnosis, and vaccines, and the data also revealed that the use of peptides in drug development was 1.26 times higher in comparison with diagnosis; however, peptides in diagnosis were 13.7 times higher than in vaccines. They also observed that the preferred diagnostic system was peptide-based enzyme-linked immunosorbent assay (pELISA), followed by microarrays and biosensors [5].

Peptides have been used successfully to detect a broad range of diseases in humans: diagnosis of cancer [22], heart disease [23], diabetes [24], Alzheimer’s disease [25], autoimmune disease [26], diphtheria toxin [27], viral [28], fungal [29] and bacterial infections [30], allergies [31], and more recently to even detect SARS-CoV-2 virus [32].

### 2.1. Peptides in Veterinary Diagnosis

On the other hand, the diagnosis of some human and veterinary diseases can be challenging considering the nature of the disease, for example in microbiology, bacterial culture is considered the gold standard, but this is difficult to perform in some cases when there are no detectable viable cells or when dealing with microorganisms with special needs like intracellular bacteria [33]. Another problem is that not all laboratories have sufficient economic resources and technology to isolate a pathogen or perform different tests to confirm its identity.

Classic serodiagnosis tests have been an aide in Veterinary Medicine to face, to some extent, these problems, and have been broadly used in the field: agar gel immune-diffusion test, enzyme-linked immunosorbent assay (ELISA), agglutinations tests, etc. are among the diverse techniques used, all of them having in common the use of serum as an initial sample; the use of some of them are approved by the World Organization of Animal Health (OIE) to identify Equine infectious anemia or the zoonotic microorganism *Leptospira* spp. [34,35] The introduction of commercial ELISA helped to accelerate massive tests to identify an etiological agent without the need to isolate it reducing the risk of working directly with the pathogen. 

Although serodiagnosis continued to be important, at some point, the methodology was quickly overtaken by fast and reliable molecular technologies that in some aspects give similar or better results in comparison [36,37]. The introduction of short peptides that mimic epitopes (mimotopes) in serodiagnosis allows keeping updated these techniques by implementing a sophisticated antigen; a small sequence that can replace a total protein but retain its antigenic capacity. Peptides have also the plasticity to be adapted to detect pathogens with different samples (total blood, tissue, or other body fluids) and not only work with serum [38].

Currently, the market of veterinary diagnosis is dominated by some well-known companies, including Zoetis, IDEXX Laboratories, Inc., and Thermo Fisher Diagnostics which offer a broad range of tests to detect pathogens; among them stand out the point-of-care tests (POCT) which are a trend that is well-received by veterinary professionals: they do not require sophisticated equipment, are user-friendly and can help to make fast decisions; interestingly, some of these companies use peptides in their POCT [39,40], but aptamers or antibodies can also be used. It is important to note that these commercial kits have variability in their specificity and sensitivity [41].

Epitope mapping constitutes a critical step to obtain adequate peptide sequences. Different approaches can be used and include the use of bioinformatics tools (in silico prediction), for this purpose software such as immune epitope database and analysis resource (IEDB) or HHpred [42,43] are available for free. These platforms are one of the most accessible options, and prediction of linear or conformational epitopes is possible, furthermore, other parameters such as secondary structures, antigenic index, hydrophilicity, flexibility can be determined [17], however, this methodology is not exempt from problems and can fail. Other options that require the use of more complex or costly technology are Pepscan, site-directed mutagenesis, mass spectrometry, and phage display, while X-ray diffraction is considered the golden standard [44].

Several decades have passed since the development of the technology to produce peptides and imaginative applications in Veterinary Medicine have been explored. Two previous reviews illustrated the scope of peptides in the diagnosis and immunologic applications in animals [17,45], however, new articles with alternative peptides that differ from the commercial kits have been published since then, and comparisons with established diagnostic systems have been performed, moreover, other systems than the classic pELISA were developed; therefore, this review aimed to update the use of peptide-based diagnostic tools in the serodiagnosis of diseases of different domestic animals and remark some aspects about their utility. The main characteristics of the diagnostic systems for peptides by species that are discussed are summarized in Table 2.

### 2.2. Swine

The porcine reproductive and respiratory syndrome virus (PRRSV) is responsible for pneumonia; abortion, and stillbirth; ewes are the major affected group. Until now it continues to be a threat in the swine industry worldwide, therefore accurate detection systems are necessary. Through the years, several pELISA had been developed with good performance, for example, a study showed that two peptides were capable to detect antibodies against this pathogen in vaccinated animals, especially one of them has a sensitivity and specificity of 97.60% and 100% relative to indirect immunofluorescence assay (IFA), moreover, the pELISA was compared with a commercial kit (IDEXX PRRSV X3 Ab ELISA kit, IDEXX, Westbrook, ME, USA), and virus neutralization test (VN). The commercial kit was able to detect more antibodies than pELISA in the first 2 weeks post-vaccination, the detection rate was similar in field serum samples, despite this, the pELISA had a good agreement with the VN test and give an economical alternative suitable for serodiagnosis [46]. 

A study carried out to detect the foot-and-mouth disease virus was performed with a peptide-based immunochromatographic test strip (lateral flow device) using colloidal gold-labeled with the peptide as the detector. The test was also compared with two ELISA probes: Ceditest^®^ (Cedi-Diagnostics BV, The Netherlands) and UBI^®^ (United Biomedical Inc., USA) for which it had an agreement greater than 90%. Interestingly, the test was able to react strongly in infected animals but not with serum samples of healthy vaccinated pigs; according to the results, the specificity and sensitivity were 100% and 95%, respectively, albeit, only serotype O was challenged, therefore further experiments with other viral strains are necessary to evaluate its usefulness [47].

Hepatitis E virus is a potential zoonotic disease that pigs carry and can be transmitted to humans on pig farms. In China, a study with synthetic peptides was compared with two commercial kits for humans, although the results can be different due to target species, the authors found that their pELISA had similar results to the recombinant protein-based kits and could be used for serodiagnosis of this disease [48].

### 2.3. Cattle

A study in Mexico developed a pELISA to detect *Anaplasma marginale*, the etiologic agent of anaplasmosis, a rickettsial pathogen. Fever, anemia, jaundice, abortion, or even death is noticeable. The use of synthetic peptides was proposed as an alternative to the crude antigen that is the actual standard. The sensitivity of the assay using a mixture of two peptides was 100% while the confidence for specificity was 95%. While it was not possible to perform a comparison to confirm no cross-reaction with serum from animals infected with *Anaplasma centrale*, an exotic microorganism in the country, there are high possibilities that the test can differentiate them [49]. 

Another tick-borne pathogen, *Babesia bovis*, was also subjected to pELISA in Mexico using the membrane antigen apical 1 (AMA-1); this protein is essential for parasite invasion of target cells, moreover it is conserved among *Babesia* isolates, which makes it an ideal candidate for diagnosis. Two peptides from this protein were found to be immunogenic in cattle (Table 2); additionally, the test was compared with IFA, and a concordance of 91.2% and 61.1% to each peptide, respectively, was observed. High sensitivity for one peptide (94.56%) but with moderate specificity (76.19%) was obtained; the second peptide had lower percentages. Nevertheless, reactivity against serum from animals in different regions of the country was noted. Modifications such as the use of several peptides from one or more proteins or changing the format of ELISA assays were suggested to upgrade the diagnosis system [50]. 

*Coxiella burnetti* (Q fever) is an intracellular microorganism that infects ruminants and causes sub-clinical diseases such as abortions, stillbirths, and reproductive disorders. The disease is widespread around the world and serological tests such as ELISA, IFA and complement fixation test (CFT) are available to detect it, however, the need of expertise and the difficulty and risk to isolate the microorganism limit their use, therefore an investigation devised a peptide-based latex agglutination test (LAT) that was further evaluated comparing with a commercial ELISA test (Biox Diagnostics, Belgium) [51]. 

While the commercial ELISA had sensitivity and specificity of 100% and 99.49%, respectively, the sensitivity of LAT was 76.19% and the relative diagnostic specificity was 75.39%; although the performance was moderate in comparison with the ELISA, the test was cheaper, fast and with potential to be worked out in field conditions. The authors considered that further research could improve the sensitivity and specificity of the developed assay, either by the selection of another peptide of the same protein or a different protein with immunogenic properties [51]. 

### 2.4. Small Ruminants (Sheep/Goat)

One pELISA was applied to detect both sheep and goat pox virus. The reason for developing this test was that low virulence strains and similarities with other diseases can be challenging, besides, the actual tests available are difficult to perform (immunofluorescence and VN test) and not readily available in different countries. The selected antigen was the structural protein P32 which is a major antigenic determinant in capripoxvirus; they established a cut-off value of 0.3 for positive samples with a specificity of 100% and a sensitivity of 95%. The authors considered that the tool developed is easier to produce and less expensive when working with herds [52].

The bacterial pathogen *Chlamydia abortus* is implicated in abortion and reproductive failure, is widely spread and is difficult to isolate, therefore, other indirect diagnosis systems are implemented including commercial and recombinant protein-based ELISA [53,54]. A study performed in Ireland contrasted three different ELISA tests (MVD-Enfer kit, UK; LSI kit, France; and a pELISA, IDvet kit, France) in ewes that received a recombinant vaccine followed by a challenge with this microorganism; the authors observed that the commercial pELISA had the lowest sensitivity between them (73.68%), a possible explanation is that the selected antigen (major outer membrane protein) could not be adequate due to the low persistence of antibodies [55].

On the other hand, an Australian study compared the same commercial pELISA (IDvet kit, France) with another kit (IDEXX, Australia), whole-cell antigen CFT, and qPCR; the research included sheep and cattle herds previously exposed to *C. abortus* and *Chlamydia pecorum*. The authors found low seropositivity of *C. abortus* with pELISA in contrast with the other tests; the results suggested false positivity due to cross-reactivity with *C. pecorum* which is endemic in Australia and low specificity in the other assays. They found that pELISA and qPCR had greater species-specificity [56]; however, more studies and a wider sample size are necessary to elucidate these disparities.

### 2.5. Equids

Equine infectious anemia (EIA) is one of the most important diseases which lack a vaccine or an effective treatment, the diagnosis is based on agar gel immunodiffusion (AGID) as the approved test, however, it is prone to false negatives and subjective readings can lead to error; this situation has been explored with other alternatives, especially ELISA [57]. For example, an investigation with pELISA reported a sensitivity of 98.6% and specificity of 95.6% when compared to AGID test and was used successfully with different equid species: horses (*Equus caballus*), donkeys (*Equus asinus*), and mules (*Equus caballus* × *Equus asinus*) [58]. Likewise, a commercial pELISA designed against the same disease (AGID-AIE Kit, LABIOFAM, Cuba) was evaluated in Mexico and similar results were obtained with a sensitivity of 100% and a specificity of 97.6%. Therefore these results in different countries suggest that pELISA is an excellent alternative to detect EIA in equid populations [57].

Another disease, the equine arteritis virus is responsible for vascular lesions, respiratory disease in adults and foals, and abortion; the pELISA was designed with two peptides of different proteins but in the end, one of them showed better discrimination capacity between positive and negative serum, additionally, the samples were characterized previously with a virus neutralization test. The study revealed a sensitivity of 95.65% and a specificity of 80.43% [59]. This pELISA and the other described in the previous paragraph were developed in Argentina.

Equine herpesvirus types 1 and 4 infect the respiratory tract and can be sub-clinical or severe are responsible for fever, lethargy, and anorexia, but type 1 is also responsible for major outbreaks with neurological disease, abortions, and perinatal foal mortality; these viruses circulate in many countries with horse populations where they can be considered endemic; additionally, due to their close genetic similarity, cross-reaction is frequent between these two types, therefore a study performed in Germany aimed to differentiate them with a pELISA; they found seroprevalences as high as 82% for type 1 and 95% for type 4 in field samples with this method; when compared with serum neutralization test, only a small percentage of samples showed discordance due to cross-reactivity [60].

### 2.6. Dogs

Ehrlichiosis is a chronic tick-borne disease in dogs whose etiologic agent is *Ehrlichia canis* and is distributed worldwide with several genotypes circulating in dog populations. A Brazilian study used synthetic peptides to detect two different genotypes in naturally and experimentally infected dogs and found 100% specificity in the ELISA, moreover, the IFA-negative dogs (negative controls) results were in agreement with the serological test. The authors concluded that the developed test was able to determine the seroprevalence of this pathogen and distinguish the genotypes even when co-infections were present in the same animal [61].

Leishmaniasis is a neglected zoonotic disease that is transmitted through a mosquito vector and is disseminated majorly in Africa, Asia, and America; in some countries, dogs are the main source of infection in urban areas, therefore, constant monitoring is necessary. A Brazilian study used a pELISA based on epitopes from A2, a stress response protein expressed during infection. The study included a commercial kit (EIE-LVC kit, Brazil) for comparison performance. The pELISA showed a better diagnostic ability with a sensitivity of 98% and specificity of 99%, while the EIE-LVC kit had a sensitivity and specificity of 90%. Another highlight was the capacity to discriminate between *Leishmania* and *Tripanosoma cruzi*-infected animals; in this aspect, the pELISA anew outperformed the kit [62].

The use of pELISA to detect this disease is particularly diverse and other serodiagnostic tests with this technology and recombinant proteins have been published through the years, which reflect the public health attention to this disease; for example, another pELISA was designed to be used in both humans and dogs, which increases its utility [63] (Table 2).

### 2.7. Cats

Feline coronavirus is a common agent in cats worldwide, infected animals suffer from self-limited enteritis, however, a small number of animals develop a more severe systemic disease known as feline infectious peritonitis (FIP) for which no effective treatment is available; one of the major problems is that ante-mortem diagnosis of FIP is challenging. The authors developed a pELISA based on epitopes from a non-structural protein with possibilities to differentiate FIP-affected animals. Two peptides were the more promising, nevertheless, a considerable variation among sera of individual cats was observed, and only one peptide was able to detect preferentially cats affected by FIP; in this case, the sensitivity was 57%, in contrast, the specificity was higher (90%). The study is the first description of a serological test that has some discriminatory power between feline coronavirus-infected cats that remain healthy and those that develop FIP [64].

### 2.8. Poultry

Previous outbreaks with H5N1 influenza virus caused serious consequences in the poultry industry and also have a high mortality rate among infected humans. The current serologic test suffers from drawbacks such as low sensitivity and cross-reactivity, thus limiting its utility; the use of a pELISA using a conserved epitope in the H5 hemagglutinin was worked out with samples of experimental animals and humans with confirmed infection; the specificity was also assessed with chicken antisera against multiples clades of H5N1 viruses, although the sample size was relatively small, the test showed no cross-reactivity and was 100% specific in the detection of H5 antibodies. The test has potential to be employed in surveillance programs in both humans and poultry [65].

Another viral pathogen that is relevant is the avian leukosis virus, animals infected show vascular and visceral neoplasms, and there is a decrease in egg production, low development, and mortality. [66] developed a pELISA to detect the avian leukosis virus, specifically subgroup J (ALV-J) which is widespread in China poultry farms, moreover they compared their in-house ELISA and a commercial kit (IDEXX ALV-J, Beijing, China) with an indirect immunofluorescence assay (IFA) as a golden standard for both tests; they found that the sensitivity of peptide-ELISA and IDEXX ELISA was 85.96% and 19.30%. The specificity of the two methods was 95.63% (175/183) and 100% (183/183), respectively. The pELISA proved to be more sensitive than the commercial kit when using sera samples [66].

Furthermore, pELISA has been evaluated as a possible alternative to the neutralization test when evaluating the immune response to the infectious bronchitis virus vaccine. The pathogen is included in the Coronaviridae family and is related to the recently emerged coronavirus SARS-CoV2. Respiratory and renal problems are observed in broiler and laying hens which generate economic losses in the poultry industry; although there are available vaccines, the immunization level should be evaluated, the pELISA showed that no cross-reactivity in immune sera against other viruses was detected, likewise, when compared with IFA the pELISA showed 98.15% sensitivity and 93.1% specificity. A positive correlation between the pELISA titers and neutralization titers suggests that this technique has the potential to replace neutralization assays when evaluating vaccines [67].

### 2.9. Other Species

A less common species of farm animal is the mink that is part of the fur industry; these mammals are prone to the Aleutian disease with symptoms such as reproductive failure, cachexia, anemia, nephritis, and even death. The disease is difficult to control; hence, rapid detection is preferable, the serological gold standard is counterimmunoelectrophoresis (CIEP) and other ELISA tests; however, drawbacks such as poor sensitivity and time-consuming are enough motives to develop an alternative with synthetic peptides. In this research, one peptide was selected as antigen and obtained sensitivity and specificity of 98% and 97.5%, respectively, the pELISA was easier to perform in comparison with CIEP and has better sensitivity that allowed it to detect the early-stage of infection [68].

Wildlife is no exception to the application of diagnostic peptides as can be seen in a study developed to detect the elephant endotheliotropic herpesvirus that severely affects young elephants, while adult specimens are carriers and spread the virus. The pELISA was compared with PCR and found that the latter was better to detect the virus in the post-mortem, sick and young animals, while the serological test surpassed the PCR to detect adult carriers. With this test, as many as 48.4% of healthy animals were positive for this pathogen [69]. The use of these innovative peptides-based tools in wildlife could help to preserve endangered species.

Interestingly, the application of peptides-based systems is not limited to just mammals and birds; their use in aquaculture species is now emerging. Kulabhusan et al., 2017 developed a peptide-based lateral flow device to detect the white spot disease in shrimp and prawns, the assay employed gold nanoparticles and was able to detect up to 12.5 µg/mL of virus protein, the sensitivity, and specificity of LFA were 100% and 97.96% 96.77%, respectively [38].

**Table 2:** Some studies using peptides to detect diseases in different animal species.

Domestic Species	Disease or Pathogen	Peptide Sequence ‡	Diagnostic System	Sensitivity/Specificity	Bibliography
Swine	Porcine reproductive and respiratory syndrome	LAPAHHVESAAGFHPITASD, VPGLKSLVLGGRRAVKRGVVNLVKYVK	pELISA Φ	97.60%/100%	[46]
	Foot-and-mouth disease	GPYAGPMERQKPLK	Lateral flow	95%/100%	[47]
	Hepatitis E	LGATSPSAPPLPPVVDLPQLGLRR	pELISA	§	[48]
Cattle	Anaplasmosis	VGDKKPSDGDID, ERSRELSRARQEDQQ	pELISA	100%/95%	[49]
	*Babesia bovis*	QEYANSTEDCAAILFDNSATDL, TAIGSPLEYDAVNYPCHIDTNGYVEPRAK	pELISA	94.56%/76.19%	[50]
	*Coxiella burnetii*	QALQKKTEAQQEEHAQQAIKENAKK	p-LAT †	76.19%/75.39%	[51]
Sheep/Goat	Sheep pox Goat pox	EAKSSIAKHFSLWKSYADADIKNSENK, FHNSNSRILFNQENNNFMYS	pELISA	95%/100%	[52]
Sheep	*Chlamydia abortus*	ID Screen^®^ Chlamydophila abortus indirect Multi-species *	Commercial pELISA	73.68%/ §	[55]
Sheep/Cattle				§	[56]
Horse/Donkey/Mule	Equine infectious anemia	KERQQVEETFNLIGCIERTH	pELISA	98.6%/95.6%	[58]
Horse		ELISA AIE-LAB^®^ kit	Commercial pELISA	100%/97.6%	[57]
Horse	Equine arteritis virus	VFLDDQIITFGTGCNDTHSVPVST, AVGNKLVDGVKTITSAGRLFSKRAAATAYKLQ	pELISA	95.65%/80.43%	[59]
	Equine herpes virus type 1 and 4	KQPQPRLRVKTPPPVTVP, TEGMKNNPVYSESLMLNV	pELISA	§	[60]
Dog	Ehrlichiosis	TEDSVSAPATEDSVSAPA, ASVVPEAEASVVPEAEASVVPEAE, HFTGPTFSEVNLSEEEKMELQEVS	pELISA	§/100%	[61]
	Leishmaniasis	MKIRSVRPLVVLLVC, PLSVGPQAVGLSVG	pELISA	98%/99%	[62]
Dog/Human	Leishmaniasis	TPAVQKRVKEVGTKP, TTVVGNQTLEKVT, VVSTSRDGTAISWK, ESTTAAKMSAEQDRESTRATLE, VGPQSVGPLSVGPQSVGPLS	pELISA	Variable (close to 100%)	[63]
Cat	Feline infectious peritonitis	PTWKFPGVKGLW, TSAKNDPWAAAV	pELISA	57%/90%	[64]
Poultry/Human	Avian influenza	CNTQCNTPMGAINSS	pELISA	100%/100%	[65]
Poultry	Infectious bronchitis	SCPYVSYGRFCIQPDGSIKQ	pELISA	98.15%/93.1%	[67]
	Avian leukosis	QALNTTLPWDPQELDILGSQ	pELISA	85.96%/95.63%	[66]
Other species					
Mink	Aleutian disease	Sequence from VP2 protein *	pELISA	98.0%/97.5%	[68]
Elephant	Elephant endotheliotropic herpesvirus	GDNDKKFSETYTKFKVYNEYERLE, ANMTKHRRKRETSSSASSK, QQHVGDPPSYDESIGSSHTYSK	pELISA	§/100%	[69]
Shrimp/Prawn	White spot syndrome	TFQAFDLSPFPS	Lateral flow	100%/97.96%	[38]

‡ Specific modifications are not included in the list, review the corresponding bibliography. Φ Peptide-based enzyme-linked immunosorbent assay. † Peptide-based latex agglutination test. * Peptide sequence not available in the article. § Data not available.

## 3. General Observations

It was evident that viral and bacterial diseases that are detrimental to the livestock industry or whose current diagnostic systems are inefficient, for example, methods that are difficult to perform or require costly equipment (culture cell, immunofluorescence), and methods that are prone to errors, were the main target of peptide-based tests to offer an alternative.

Many studies with peptides that were reviewed included commercial kits or officially approved tests by federal governments and international organizations such as the World Organization for Animal Health (OIE) [70] to know their performance in comparison with these well-established systems; the results show that most of them have similar performance, some even outperformed them and others were less efficient than their counterparts. If the peptide test is good enough it could substitute some more expensive kits.

Almost all the diseases listed in Table 2 have commercial kits that can include POCT, ELISA (recombinant proteins), or real-time PCR, however, not all of them are available in different countries or are relatively expensive, therefore, the investigations that were reviewed allow the use of in-house tests when there are limitations to acquire a commercial product. Moreover, there are a large number of investigations about epitope mapping of more diseases that disclose potential peptides to be used as diagnostic tools or vaccines [71,72], albeit, they are not being put in practice with field samples; a possible explanation is that the early use of recombinant proteins and commercial kits has generated a habit, e.g., IDEEX has been in the market since 1987 [40]; furthermore, the use of a trademark generates trust in the researcher or clinical veterinarian and they are user-friendly which makes them more attractive. These traits should be incorporated into new peptides to offer a flashier alternative, especially exploring the possibility of being adapted in POCT which is nowadays the state-of-the-art in fast diagnosis.

The peptide-based systems reviewed in this study showed their capacity and flexibility to expand or reduce their target according to what the researcher is looking for: the tests could be applied in one or more species, while others are able to detect a specific variant or genotype, furthermore, a few of them could differentiate between vaccinated and natural-infected animals, and they can also be suited to evaluate the performance of vaccines post-inoculation, in order to know an approximate time when antibodies are detected.

Although their use in diagnosis has expanded considerably, the application of other devices different from pELISA such as microarrays and lateral flow devices are relatively less common, possibly due to the ease and practicality of ELISA. Additionally, more comparisons in different circumstances are needed to assess their utility, and finally, the marketing of diagnostic devices remains relatively unexplored and is an interesting field to be covered.

## 4. Peptide Synthesis Approaches

Peptide synthesis can be achieved by three different approaches: (a) chemical synthesis; (b) recombinant DNA technology and (c) enzymatic synthesis [73].

Chemical synthesis which is the most popular method has two procedures to obtain peptides; one involves the use of solution-phase synthesis (SPS), however, this method is tedious and time-consuming since isolation and characterization should be performed after each step, therefore, solid-phase peptide synthesis (SPSS) has been preferred since its creation, however, SPS continues to be used for short peptide chains (typically less than 10 amino acids) and is used in large-scale production; even now technique improvements are being made. It must be noted that both methods can be combined [9,74,75,76].

Regarding the history of SPSS, it is attributed to Robert Bruce Merrifield who first developed the methodology in 1963 [77]. One of the most important improvisations was the introduction of a 9-fluorenylmethoxycarbonyl group (Fmoc) in 1970 by Han and Carpino as a new base-labile protecting group of amines, this greatly contributed to establishing the actual approach that is the most common strategy of SPSS nowadays [74,78,79].

The principle of SPPS is based on attaching the first amino acid to a resin, then proceeding with peptide chain elongation to ultimately provide the target peptide. A resin is composed of polymeric solid support linked permanently to a linker (bifunctional spacer, or handle) that facilitates temporary anchoring of the first amino acid to the polymeric support, one of the most common solid support used is 1–2% divinylbenzene-cross-linked polystyrene in the form of a bead with diameters of about 50 microns, although others supports are feasible [79,80,81].

Even if the method has improved a lot in the last years, limitations and technical intrinsic problems remain, for example, the synthesis of ~50 amino acids or more and the difficulty of synthesizing hydrophobic peptides [82]. The demand for huge amounts of synthetic peptides for pharmaceutical applications represents a challenge to satisfy the market, the use of large-scale jacketed solid-phase reactors is necessary when dealing with quantities >100 kg [83]. Another point of concern is the substantial use of organic solvents such as dimethylformamide (DMF) and other compounds that are toxic to the environment and human beings; several green options are being investigated [84,85].

The principle of recombinant synthesis is to make use of the natural production machinery of the cells. The use of recombinant technologies offers cost-effective means for large-scale peptide manufacture, nevertheless, it is not free of particular problems to express and purify peptides. Both prokaryotic and eukaryotic host systems can be used to produce therapeutic protein/peptides that include cell cultures of mammals (for example Chinese hamster ovary cells), insects, plants, molds, and bacteria such as *E. coli*, *Pseudomonas fluorescens*, *Bacillus subtilis*, *Staphylococcus carnosus*, *Lactococcus lactis*, *Sacharomyces cerevisiae*, and *Pichia pastoris* [9,86,87,88,89].

Despite the success of recombinant production of peptides, chemical synthesis is the prevalent manufacturing approach. Two reasons can explain this situation: the first is related to the nature of the biotechnological process itself. To obtain a recombinant peptide a complex process is required (selection of a suitable expression system, construction of expression vector, bioprocess development in the bioreactors at different scales, and ending with downstream processing), therefore, is labor-intensive and takes a substantial amount of time to develop and market in comparison to chemical synthesis; notwithstanding, attractive features such as the synthesis of larger peptides, relative large-scale production, and environmentally friendly synthesis make this technology a good option. These and other features including advantages and drawbacks of peptide production by recombinant technology are discussed previously [9].

The enzymatic synthesis makes use of different enzymes of animal, vegetable, and microbial origin, the vast majority of these enzymes are proteases and transpeptidases, for example, cysteine proteases (papain, bromelain), serine proteases (α-chymotrypsin, proteinase K, trypsin, subtilisin) and esterases (lipase) [75,90,91]. In comparison with SPSS technology alone, where it is challenging to produce longer peptides, enzymes offer the opportunity to ligate several smaller peptide fragments, with excellent region and chemoselectivity and perform the catalysis of reactions under mild conditions. The use of both chemical and enzymatic synthesis (chemo-enzymatic peptide synthesis) is an attractive option for researchers as it could enhance the flexibility to synthesize peptides [73].

Another advantage of enzymatic synthesis is that it is environmentally friendly, avoiding the need to use harmful solvents; the drawbacks, however, are low product yield and non-ideal purity [73,92]. Despite these limitations, the methodology allows to process plant and animal protein hydrolysates that provide highly digestive peptides and bioactive peptides, therefore it is a valuable tool for animal nutrition [93].

## 5. Advantages and Drawbacks of Synthetic Peptide-Based ELISA

Like each method, the application of ELISA for diagnostics, as well as synthetic peptides have their traits, therefore, the optimization of each ELISA protocol must consider the most suitable peptide and the pertinent modifications in its structure.

ELISA has been a well-known method for several decades, the use of direct, indirect, or sandwich ELISA has been described elsewhere, and every one of them needs to be optimized; fortunately, this is facilitated thanks to the use of a chessboard titration, troubleshooting guides and some tips [94,95,96]; however, the particularities of synthetic peptides should be added and several strategies are feasible to overcome these problems.

Among the most common drawbacks responsible for poor peptide performance is the peptide length (usually when it is shorter than 20 residues), this limits the number of sidechains available for both adsorption and target recognition; howbeit, several improvements have been described and include modifications of the microtiter plate polystyrene surface, some of them coating it with maleimide-activated bovine serum albumin, maleimide-activated keyhole limpet hemocyanin or alcian blue [97,98,99]. Other carriers include the use of biotinylated peptides that take advantage of the strong bond between biotin-streptavidin or albumin-conjugated peptides [100,101,102].

One more limitation is that almost all of the petides used as mimotopes are linear; conformational epitopes cannot be synthetized with the actual technology, this is expected as three dimensional structures are more complex, however other approaches of peptides synthesis enable the use of dendrimeric peptides which have increase bio-availability and have been used in veterinary serodiagnosis studies with promising results. It is possible that dendrimeric peptides could increase the sensitivity and specificity more than actual linear peptides [103,104].

Moreover, some investigations have focused on enhancing the peptides-polystyrene adherence, some of them using a polystyrene-binding sequence (PS-tag) which deploys high affinity toward polystyrene, such a sequence is then attached to the target peptide increasing the binding capability in comparison with native peptide or other modifications such as biotin-tagged peptides [105,106]. They all seek to increase the peptide adsorption rate and functionality.

But why do synthetic peptides have more advantages over recombinant proteins? Although it cannot be true in all cases it provides several benefits:

- Allow the construction of peptides that are incompatible with recombinant methods
- Possibility to customize peptides and aggregate other molecules
- It is an alternative for laboratories that do not have the technology for recombinant processes as the sequences are processed by a company
- Shorter time to obtain a peptide
- Easy to manipulate (powder)
- Fewer possibilities of cross-reaction with other similar pathogens or capacity to differentiate variants within the same microorganism

Although is evident that peptide-based systems are an interesting and functional alternative, their capability must be compared with other known tests to ensure that it is the best choice available for each particular disease; recombinant proteins in serodiagnosis or qPCR continue to be excellent options with its peculiarities that according to the situation have adequate performance. Ultimately, cost, performance, and suitability should be taken into account when selecting the diagnostic system.

## 6. Conclusions

We reviewed 23 studies that used synthetic peptides and were applied to detect 21 different diseases in 10 domestic animals and four non-domestic animals; It is important to highlight that almost all of them differ from the commercial kits and reflect the opportunity to develop in-house protocols. Viral and bacterial diseases with high morbidity and mortality in animal production were the main goal of these investigations, which have also diagnostic flaws with other methods such as low sensitivity or specificity, reproducibility, or are difficult to perform. Moreover, two studies also used human serum for some zoonotic diseases of concern; this is interesting because the same diagnostic system can have multi-species applications which is a feature of several commercial devices and could compete with them.

According to the diagnostic system, pELISA was the most used with 19 publications, followed by lateral flow devices and peptide-based latex agglutination tests. Almost all of the investigations developed their own diagnostic devices and only three investigations purchased available commercial peptides; the low amount of marketed pELISA could be because recombinant proteins are more commonly used in ELISA, however, it is expected in the next years that an increasing number of new peptide drugs, AMP, and other products for pathogenic and non-pathogenic diseases will be patented and marketed worldwide. Every year, new peptides are isolated from animal sources and their therapeutic potential is explored with promissory results.

Regarding the technology used to obtain peptides, synthetic peptides offer an available option to create peptides more easily; new strategies for large peptides and difficult sequences are being explored and are the principal focus of several investigations and could be an important step in peptide synthesis. Despite the fact that we encompassed more research with synthetic peptides, the use of recombinant DNA technology remains well-established support in Veterinary Medicine when dealing with protein, and new protocols for peptide synthesis are being developed, hence it is expected that recombinant peptides continue to be another viable option.

Among the things that need to improve is the inclusion of other epitope mapping methods and comparative studies with other molecular methods. Additionally, a broad spectrum of marketed peptide diagnostic systems is necessary to attract more researchers and government agencies; the introduction of POCT with peptides could be interesting and could compete with already established products. The use of these in-house peptides is incipient in some cases, hence, it is necessary to increase the number of samples and obtain more tangible results about sensitivity, specificity, accuracy and other parameters that lack some studies (Table 2) to make them more reliable.

Finally, the contrast with other tests confirms the sturdiness and validity of peptides-based systems, especially for pELISA; therefore, synthetic peptides are an alternative option with the possibilities of facilitating the diagnosis of some diseases, however, more large-scale studies are necessary and complementary analysis to approve a generalized use of these diagnostic tools with the potential to be employed in national programs of eradication, control, or vaccination.

## Figures and Tables

**Table 1:** Some peptides for Veterinary use approved by the Food and Drug Adminstration.

Drug Name/ Active Ingredient	Indication
Oxytocin Injection Oxytocin	Uterus atony, retained placenta, postpartum agalactia
OvuGel™ Triptorelin acetate	Synchronization of ovulation in weaned sows and gilts
Ovuplant™ Deslorelin acetate	Control and synchronization of the estrous cycle and ovulation induction in mares
P.G. 600^®^ Chorionic Gonadotropin	Stimulation of follicles development and their ovulation in gilts and sows
Granulex^®^ V Trypsin	External wounds (wire cuts, lacerations, abrasions), removal of dead tissue and debris
FOLLTROPIN^®^ porcine pituitary follitropin	Induction of superovulation in beef and dairy heifers and cows
ProZinc^®^ Insulin	Diabetes in dogs and cats
Posilac™ n-methionyl bovine somatotropin	Increment in milk production in dairy cattle
Imrestor™ Pegbovigrastim, an analog of recombinant endogenous bovine granulitecolony stimulating factor (bG-CSF)	Promote neutrophil proliferation and reduce mastitis risk in dairy cattle
Improvest^®^ gonadotropin factor analog-diphtheria toxoid conjugate	Immunological castration (temporally suppression of testicular and ovarian function) of pigs

## Data Availability

Not applicable.
